# The Role of Viral Pathogens in the Promotion of Epithelial–Mesenchymal Transition and Neoplastic Cell Invasion

**DOI:** 10.3390/v18101156

**Published:** 2026-10-18

**Authors:** Sharof Tugizov

**Affiliations:** Department of Medicine, School of Medicine, University of California–San Francisco, San Francisco, CA 94143, USA; sharof.tugizov@ucsf.edu

**Keywords:** virus, neoplastic cells, epithelial–mesenchymal transition, cancer invasion

## Abstract

Epithelial-to-mesenchymal transition (EMT) is a multistep process in which stationary, polarized epithelial cells lose cell-to-cell adhesion and acquire a mesenchymal phenotype with migratory and invasive properties. Migrating EMT cells express both epithelial and mesenchymal markers, an intermediate hybrid state that allows them to retain some epithelial traits for survival while gaining mobility. After migration and metastasis to a secondary tumor site, EMT cells revert to an epithelial-like state via a mesenchymal-to-epithelial transition (MET) to establish new tumors. Cells undergoing EMT may also acquire cancer stem cell properties, leading to chemotherapy resistance and cancer recurrence. Accumulating evidence suggests that both oncogenic and non-oncogenic viruses can induce EMT. Among the oncogenic viruses, EMT has been reported in neoplastic cells infected with human papillomavirus, hepatitis B and C viruses, Epstein–Barr virus, and Kaposi sarcoma-associated herpesvirus. Non-oncogenic viruses, including human cytomegalovirus and human immunodeficiency virus, can also induce EMT in neoplastic cells. Furthermore, coinfection of neoplastic cells with two or more oncogenic viruses or oncogenic and non-oncogenic viruses may lead to synergistic and/or additive effects with respect to EMT induction. This review presents our current understanding of the molecular mechanisms underlying virus-induced EMT and the role of this transition in promoting neoplastic disease.

## 1. Introduction

Oncogenic viruses, including human papillomavirus (HPV), hepatitis B virus (HBV) and hepatitis C virus (HCV), Epstein–Barr virus (EBV), and Kaposi sarcoma-associated herpesvirus (KSHV), play critical roles in the development of numerous human cancers of epithelial origin. Virus infections are directly responsible for ~12% to 20% of human cancers worldwide. High-risk HPVs cause 99% of all cervical and anal cancers; likewise, 70–80% of all cases of hepatocellular carcinoma (HCC) are linked to HBV/HCV infection [1,2,3,4]. Viral oncogenes may alter, reprogram, or hijack the physiologic functions of cellular proteins, thereby driving the proliferation and invasion of neoplastic cells. Among the mechanisms associated with these processes, many viruses induce epithelial– or endothelial–mesenchymal transition (EMT or EndMT, respectively), which are processes that facilitate migration and invasion of neoplastic cells [5,6,7,8,9].

Although they do not support classical viral oncogenesis, non-oncogenic viruses may also induce EMT. Recent evidence suggests that human cytomegalovirus (HCMV) and human immunodeficiency virus (HIV) may contribute to the proliferation, migration, and invasion of neoplastic cells through the induction of EMT and other mechanisms [7,10,11,12,13,14,15,16,17,18,19,20].

Viral proteins can activate signaling pathways, including those involving transforming growth factor-beta (TGF-β), mitogen-activated protein kinases (MAPKs), Notch, Wnt/β-catenin, and nuclear factor kappa-light-chain-enhancer of activated B cells (NF-κB) [21,22]. Virus-mediated activation of these pathways upregulates the expression and activation of specific transcription factors, including zinc finger E-box-binding homeobox (Zeb) 1 and 2, basic helix–loop–helix twist homolog (Twist) 1 and 2, and zinc finger Snail proteins 1 (Snail) and 2 (Slug), leading to the induction of EMT [21,22]. Viral protein-induced EMT may accelerate neoplasia by allowing stationary epithelial cells to adopt invasive mesenchymal traits, thereby promoting metastasis, stemness, and therapeutic resistance. Pro-inflammatory cytokines interleukin (IL)-1), IL-6, and tumor-necrosis factor alpha (TNF-α) produced during viral infection, can also lead to EMT, as these mediators can also activate transcription factors and signaling pathways that cause epithelial cells to lose their adhesive properties [23,24].

In this review, we consider the mechanisms underlying virus-induced EMT and the contributions of this phenotypic change to the progression of neoplastic diseases.

## 2. Epithelial–Mesenchymal Transition

Epithelial–mesenchymal transition (EMT) is a dynamic, physiologic process that regulates the differentiation of cell lineage identity during embryogenesis [25] and also plays a critical role in wound repair and tissue remodeling [26,27]. EMT also contributes to critical neoplastic processes [28,29] (Figure 1). Neoplastic epithelial cells undergoing EMT lose cell–cell junctions and apical–basal polarity [30]. As a result, these cells lose their adhesive properties and escape from the primary tumor, enter circulation, and initiate the metastatic cascade [31,32,33,34,35].

EMT can be initiated in cancer cells by activation of the TGF-β signaling pathway [36] (Figure 2). Ligand binding to TGF-β receptors (TGF-β R1/2) leads to phosphorylation of transcription factors SMAD2/3, which then form complexes with SMAD4. These complexes translocate into the nucleus, leading to upregulation of Snail, ZEB, and Twist family transcription factors resulting in downregulation of E-cadherin and upregulation of vimentin, fibronectin, and N-cadherin expression [37,38,39]. Upregulation of fibronectin and N-cadherin is critical for neoplastic cell invasion [40,41]. Snail activation also inhibits the expression of the tight junction proteins, claudins and occludin, which ultimately leads to epithelial cell depolarization and EMT [42].

TGF-β upregulates and activates numerous transcription factors, including Forkhead box protein C2 (FOXC2), which drives EMT by incorrectly localizing membrane-bound E-cadherin to the cytoplasm and upregulating mesenchymal markers [43]. TGF-β also promotes the expression of Goosecoid, which cooperates with FOXC2 to maintain the mesenchymal state [44]. TGF-β/SMAD-activated SMAD-Interacting Protein (SIP1) (also known as ZEB2 or ZFHX1B) binds directly to the regulatory domain of the E-cadherin gene to repress its transcription [45]. SIP1 also suppresses the expression of other critical cell-to-cell junction components (e.g., tight junctions [claudins, ZO-3] and desmosomes [desmoplakin]), and thus contributes to EMT induction by dismantling tight/adherens junctions [46].

TGF-β also activates other intracellular signaling cascades, including PI3K/AKT, MAPK (ERK1/2, p38, JNK), and RhoA, which collectively cooperate with the aforementioned SMADs to promote EMT [47,48,49]. PI3K/Akt signaling induces EMT by upregulating EMT-specific transcription factors Snail and Twist and cytoskeletal reorganization [50]. Similarly, the TGF-β-activated MAPK signaling pathway also induces EMT via phosphorylation of Smad2/3 and Twist1 [47,48]. As noted by Chapnick and colleagues [51], crosstalk between the TGF-β and MAPK signaling cascades is important for the induction and maintenance of the EMT phenotype.

The Wnt/β-catenin signaling pathway (Figure 2) plays a major role in the induction of EMT, facilitating β-catenin translocation to the nucleus, where it activates Snail, Twist, and ZEB1/2, suppresses E-cadherin, and upregulates N-cadherin and vimentin expression [52,53]. Overexpression of Wnt ligands (e.g., Wnt1) can enhance EMT and facilitate the detachment of cancer cells from primary tissues [54,55].

Neurogenic locus notch homolog protein (Notch) signaling induces EMT when Notch ligands delta or serrate/Jagged bind to their receptor (Figure 2). This interaction leads to the translocation of the Notch intracellular domain into the nucleus, where it activates Snail and initiates EMT [56].

Hedgehog signaling also induces EMT via upregulation of Snail, Twist, and Zeb-1 [57,58]. Hedgehog ligands (e.g., Sonic Hedgehog) bind to the surface receptor Patched, thereby relieving inhibition of the Smoothened (SMO) coreceptor [59]. Activated SMO triggers an intracellular cascade that results in the nuclear translocation of glioblastoma family transcription factors, which, as a group, downregulate E-cadherin and upregulate mesenchymal markers N-cadherin, vimentin, and fibronectin [60].

NF-κB signaling is also an important contributor to EMT induction. Activation of NF-κB and translocation of p65 into the nucleus lead to Twist1, ZEB1, and ZEB2 binding, which decreases E-cadherin expression and increases the expression of mesenchymal markers N-cadherin and vimentin [61,62].

Finally, the PI3K/AKT signaling pathway also contributes to the induction of EMT by suppressing epithelial and upregulating mesenchymal markers. Activation of PI3K/AKT upregulates the expression of transcription factors that drive EMT, including Snail, Slug, Twist, and Zeb1 [50,63]. PI3K/AKT signaling also suppresses GSK-3β kinase activity via phosphorylation at serine 9 (Ser9). This leads to the stabilization of Snail and Slug and promotes EMT [50,64].

Crosstalk between different signaling pathways, including TGF-β, Wnt/β-catenin, Notch, and Hedgehog, leads to mutual amplification, increased activation of Snail, Slug, and Twist, and a stable mesenchymal state in tumor cells [57,65]. These cooperative interactions create feedback loops that efficiently drive EMT in target neoplastic cells [66].

Receptor tyrosine kinase (RTK) activation triggers EMT by binding extracellular ligands (e.g., epidermal growth factor, fibroblast growth factor, platelet-derived growth factor) (Figure 2). Ligand binding leads to receptor dimerization and trans-phosphorylation [63,67], activating downstream MAPK and phosphoinositide 3-kinase (PI3K) pathways, and ultimately promoting EMT [63].

Proinflammatory cytokines, e.g., tumor necrosis factor-alpha (TNF-α), IL-1, and IL-6, also upregulate transcription factors, including Snail and ZEB1/2, by activating NF-kB and MAPK signaling, thereby inducing EMT [24,68]. IL-6 also induces EMT by binding to cell-surface receptors and activating JAK/STAT signaling [69]. Activated JAKs phosphorylate STAT3, which then translocates into the cell nucleus. Nuclear STAT3 acts on SNAIL and TWIST, resulting in activation of mesenchymal genes and suppression of epithelial E-cadherin expression [70]. TNF-α activates NF-κB to induce COP9 signalosome 2, which blocks the ubiquitination and degradation of the Snail protein, maintaining it in a stable state, facilitating ongoing repression of epithelial genes and promoting EMT [71].

Cells undergoing EMT express and release matrix metalloproteinases (MMPs), which degrade the extracellular matrix, facilitating cell invasion and migration. MMP-2 and MMP-9 degrade cell-to-cell junctions and structural barriers, for example, the basal lamina [72,73]. Moreover, MMPs also activate TGF-β and Wnt/β-catenin signaling pathways and can induce Snail expression, promoting the EMT program [72].

EMT is regulated by epigenetic modifications (e.g., DNA methylation, histone modifications, and activation of non-coding RNAs [ncRNAs]) that coordinate widespread changes in gene expression without altering the underlying DNA sequence. DNA methyltransferases methylate the cytosine- and guanine-rich promoter region of the E-cadherin (*CDH1*) gene, suppressing protein expression and inducing EMT [74,75]. Histone modifications regulate EMT by altering chromatin structure and recruiting transcription factors that silence epithelial genes or activate mesenchymal genes. For example, histone deacetylase 1 (HDAC1) and HDAC2 remove acetyl groups from histone tails, tightening DNA-histone binding and preventing the transcription machinery from accessing epithelial genes [76]. Specific ncRNAs regulate EMT by targeting master transcription factors, acting as competing endogenous RNAs, and modulating critical signaling pathways, e.g., TGF-β and Wnt/β-catenin [77]. MicroRNAs (miRNAs) (e.g., the miR-200 family) form double-negative feedback loops with EMT transcription factors (e.g., ZEB1/2) to regulate EMT and maintain cell plasticity [78].

EMT is a multistep process. Cells in intermediate stages may express both epithelial (E-cadherin) and mesenchymal markers (vimentin). This state, known as partial EMT or hybrid EMT phenotype, contributes significantly to the invasiveness of cancer cells by promoting collective cell migration and rendering cells resistant to drug treatment and radiotherapy [32,33,34,35] (Figure 3). Hybrid EMT may also express stem cell markers and become cancer stem cells (CSCs) with undifferentiated stem cell properties and the capacity for self-renewal and uncontrolled proliferation [32,33,79,80,81,82,83,84]. Symmetrical or asymmetrical division of CSCs can generate undifferentiated stem cells for self-renewal and differentiated CSCs that contribute cells that can proliferate, invade other tissues, and resist chemotherapeutic agents [85,86,87].

Cancer EMT cells from primary tumors enter the circulation (blood or lymph; intravasation) [88]. EMT cells can ultimately migrate to a new tissue or organ (extravasation), and revert to a state characterized by the expression of epithelial markers (Figure 4). This process, known as mesenchymal–epithelial transition (MET), is a critical feature of tumor metastasis, colonization, and formation of secondary tumor lesions [89,90]. Neoplastic cells under the MET program are less mobile, upregulate adhesion proteins, and form stationary tissue layers. EMT and MET are reversible biological processes that allow cells to shift between a non-invasive, stationary, epithelial phenotype and a mobile, invasive mesenchymal phenotype.

## 3. Oncogenic Viruses

### 3.1. Human Papillomavirus

HPV is an oncogenic virus of the *Papillomaviridae* family that infects oropharyngeal and genital mucosal epithelial cells. Multiple high-risk oncogenic HPVs, including HPV16 and HPV18, have been identified as the etiological agents of many oropharyngeal and almost all anogenital cancers [91,92]. HPV oncoproteins E6, E7, and E5 play critical roles in the initiation and progression of HPV-associated neoplasia [91,92].

Development of HPV-associated neoplasia is a multistage process, beginning with the initial appearance of low-grade and high-grade premalignant epithelial lesions, and their subsequent development into invasive cancer [93,94]. HPV-infected premalignant lesions may persist for many years without EMT induction [94,95], and the majority of these lesions (70–95%) ultimately undergo regression [96,97]. Induction of EMT in HPV-infected high-grade lesions accelerates the progression of premalignant cells into invasive cancer [95].

EMT plays a significant role in neoplastic cell invasion in high-risk HPV-infected genital cancers [86,87,98,99,100]. HPV-infected cervical cancers acquire the mesenchymal phenotype, notably a reduction in E-cadherin expression and induction of vimentin [99,100].

High-risk HPV oncoproteins E6, E7, and E5 activate TGF-β signaling, and upregulate Twist, Zeb, and Slug transcription factors that promote EMT [95,101,102,103,104,105,106,107,108]. Morgan and colleagues [109] reported that the high-risk HPV E6 protein activates JNK and epithelial growth factor receptor (EGFR) signaling and induces EMT by upregulating Slug and vimentin.

HPV-16 and HPV-18 infection in cervical cancer cells led to the induction of EMT by upregulating calcium signaling mediator transient receptor potential canonical-3 (TRPC3) expression, leading to EMT via activation of MAPK signaling [110] (Table 1). HPV-16 E5 and E6/E7 proteins activate MAPK via upregulation of EGFR [111,112,113,114,115,116] and enhanced ZEB1, Snail1, Slug, and Twist1 expression, thereby inducing EMT [95,117]. HPV-16 E5 and E6/E7 proteins also activate PI3K/AKT signaling, and upregulate expression of Snail, Slug, Twist, to promote EMT [63,118,119]. Cheng and colleagues [120] found that HPV-16 E6/E7 promoted fibroblast growth factor 2 and 4 expression in cervical cancer cells, leading to cell invasion via upregulation of vimentin and downregulation of E-cadherin. Likewise, Jung and colleagues [121] correlated HPV-16 infection in head and neck cancer with Zeb expression and induction of EMT.

Veeraraghavalu and colleagues [122] reported that HPV 16 E6/E7 induced Jagged1 expression in cervical tumors. Results from in vitro experiments revealed that E6/E7-mediated increases in Jagged1 expression activated PI3K signaling and induced EMT. Given that Jagged1 is a ligand for Notch receptor, increased expression of this ligand may activate Notch signaling, which is also critical for EMT induction.

High-risk HPV E6 and E7 oncoproteins directly and indirectly activate the PI3K/AKT/mTOR cascade [123], resulting in the increased expression of the immune checkpoint protein programmed death-ligand 1 (PD-L1) and EMT [124,125]. Thus, HPV E6/E7 upregulate both PD-L1 and promote EMT to facilitate both immune evasion and cancer cell invasion [121,126,127].

Expression of the HPV oncoproteins E6/E7 in normal foreskin keratinocytes resulted in a spindle-like morphology associated with reduced expression of E-cadherin and induction of vimentin and fibronectin [108]. High-risk HPV E6 and E7 oncoproteins induced matrix metalloproteinase (MMP)-2 and MMP-9 expression. These enzymes cleave E-cadherin, leading to loss of adherens junctions and the initiation of EMT [128,129,130,131,132,133,134]. The presence of high-risk HPV E6/E7 also resulted in the increased expression of membrane type 1-matrix metalloproteinase (MT1-MMP), an enzyme that digests the extracellular matrix and thus leads to the increased migratory potential of EMT cells [131,135,136,137,138,139]. Treatment of HPV16-immortalized cervical epithelial SiHa cells with TGF-β leads to EMT and increased invasiveness [140], suggesting that TGF-β-mediated signaling may synergize with HPV E6/E7-induced EMT.

HPV-16 E5 upregulates EGFR-linked signaling pathways, including MAPK/ERK and PI3K/Akt, downregulates E-cadherin, and promotes cellular motility [141]. Furthermore, E5 promotes EMT by downregulating epithelial-specific fibroblast growth factor receptor 2 (FGFR2b) and upregulating mesenchymal-specific FGFR2c [142].

### 3.2. Epstein–Barr Virus

EBV, also known as human herpesvirus 4, is a very common, contagious virus of the Herpesviridae family, affecting approximately 90% people globally. EBV is the primary cause of infectious mononucleosis (i.e., “mono” or the “kissing disease”) and spreads mainly through saliva and similar shared items [143]. While most infections are harmless or asymptomatic, EBV is a classified oncogenic virus, as it has been linked to lymphomas, nasopharyngeal cancer, and stomach cancers [144].

EBV Latent Membrane Protein 1 (LMP1) is a viral oncoprotein that induces epithelial cell EMT, particularly in nasopharyngeal carcinoma (NPC) [145,146] (Table 1). The results of in vitro experiments have shown that EBV LMP1 drives this process by activating NF-κB, ERK-MAPK, PI3K/Akt, and TGFβ signaling pathways, leading to reduced E-cadherin, increased vimentin, enhanced cell migration, and invasion [147,148,149]. EBV LMP1-mediated activation results in the upregulation of Snail and Twist and induction of MMP-9 expression, promoting the invasion of neoplastic NPC cells [150,151,152,153].

EBV LMP1-activated PI3K/mTOR/Akt signaling also initiates EMT induction and generation of cancer stem cells, which accelerates their tumorigenic and metastatic features [146,153,154]. Both mTOR protein complexes, mTORC1 and mTORC2, promoted cell migration and invasion in NPC cells, suggesting that mTORC1/2 are co-regulators of NPC metastases [146,154].

EBV latent membrane protein 2A (LMP2A) induces EMT in NPC cells by activating the PI3K/Akt signaling cascade [155], thereby promoting NPC cell migration, invasion, and acquisition of stem-like properties that contribute to tumor recurrence and metastatic disease. LMP2A also enhances mTOR activation, thereby increasing the expression of metastasis-associated protein 1, which promotes EMT [156]. Expression of LMP2A in NPC cells increases the number of cancer stem cells, facilitating self-renewal and enhancing tumor growth [155].

EBV LMP1 activates PD-L1 expression and promotes EMT in neoplastic cells [152,157]. LMP1 targets major oncogenic and inflammatory signaling pathways, including JAK/STAT, PI3K/Akt, and MAPK, thereby driving PD-L1 expression on neoplastic cell surfaces and promoting cancer cell evasion of the immune response [157]. LMP1 also promotes the expression of the transcription factors Snail and Twist, thereby inducing EMT and cancer cell invasion [152].

Co-expression of both EBNA1 and EBNA3C in cancer cells can induce EMT by upregulating Slug, Snail, Zeb1, and vimentin; this leads to the disruption of the tight junction protein ZO-1, downregulation of the cell adhesion molecule E-cadherin, and nuclear translocation of β-catenin [158].

EBV encodes numerous miRNAs, which are posttranscriptional regulators that promote EMT and accelerate invasion and metastasis in NPC and gastric cancer [159,160,161]. Downregulation of members of the miR-200 family (miR-200a, miR-200b) induces EMT in EBV-associated gastric carcinoma cells and promotes cancer metastasis [162,163]. EBV infection leads to decreased expression of these miRNAs, resulting in the activation of Wnt/β-catenin and c-Jun/Slug signaling, upregulation of ZEB1/ZEB2, and thus increased EMT and tumor invasion [159,160,161,164]. EBV nuclear antigen 1 (EBNA1) induces EMT, promoting metastasis and invasion of NPC cells by activating TGF-β signaling and inhibiting miR200. This results in the upregulation of Zeb1 and Zeb2 transcription factors, E-cadherin downregulation, and upregulation of mesenchymal markers [165].

### 3.3. Hepatitis B Virus

HBV is an enveloped double-stranded DNA virus that replicates in liver cells [166]. HBV enters cells via the actions of its surface glycoproteins (HBsAg). HBV also has a core protein (HBcAg) that it uses to build its capsid, a polymerase that is critical for genome replication, and a regulatory X protein (HBx) that promotes transcription of viral genes [166,167,168].

HBV causes acute or chronic liver disease, which may develop into cirrhosis and hepatocellular carcinoma (HCC). HBV is considered a grade 1 carcinogen and may also increase the risk of bile duct and gastric cancers, as well as non-Hodgkin lymphoma [166,167,169].

During the years 2022 to 2025, approximately 250 to 300 million people were living with chronic HBV infection worldwide, a condition that led to over 1 million deaths annually from cirrhosis and liver cancer [170,171]. HBV causes roughly 37.5–39% of all liver cancers, with over 200,000 new cases of HBV-related liver cancer reported in 2021 alone [170,171].

The HBx protein induces EMT in hepatocytes, initiating cancer cell migration, invasion, and liver fibrosis [172]. HBx promotes EMT by activating PI3K/AKT, JNK, and c-Src signaling pathways that downregulate E-cadherin and elevate vimentin expression, thereby driving HBV-associated HCC progression [172,173,174] (Table 1).

The HBx protein reduces E-cadherin expression via induction of the transcriptional repressors E12/E47 and Snail, thereby promoting neoplastic cell detachment [175]. HBx activates the PI3K/Akt/GSK3β pathway, which stabilizes Snail [172,173,174]. HBx also activates c-Src kinase to activate vimentin expression and induce the characteristic spindle-like mesenchymal morphology [172,173,174].

HBx and HBc proteins induce autophagy via the JNK pathway, which also contributes to EMT and liver fibrosis [176]. Zhu and colleagues [177] reported that HBx upregulates transcription factor ASCL1, which promotes von Willebrand factor transcription, leading to tumor cell EMT, proliferation, migration, invasion, and reduced apoptosis.

HBV infection induces EMT by upregulating oncogenic miR-520c-3p and downregulating tumor-suppressive miRNAs miR-148a and miR-122 [178,179,180]. HBx also suppresses miR-148a and reduces the expression of hematopoietic pre-B cell leukemia transcription factor-interacting protein, thereby inducing EMT and metastasis via Akt/ERK signaling pathways [179]. Liu and colleagues [181] found that HBV infection suppresses miR-15a, leading to upregulation of Smad7 and induction of EMT. HBsAg upregulates the long non-coding RNA LINC00665 by activating NF-κB signaling, leading to reduced miR-126-5p activity [182,183] and increasing vascular endothelial growth factor A expression, thereby promoting both angiogenesis and EMT [183].

### 3.4. Hepatitis C Virus

HCV is an enveloped RNA virus that infects liver cells and causes both acute and chronic infections, leading to cirrhosis and cancer [184,185]. Approximately one million new HCV infections occur yearly worldwide. In 2022, more than 50 million people were living with chronic HCV infection, causing ~242,000 deaths annually, primarily attributable to cirrhosis and HCC [186,187].

HCV’s 10 key proteins are generated by cleavage of a single polypeptide, including 3 structural proteins (a core and glycoproteins E1 and E2 that facilitate virus entry) and 7 nonstructural proteins (p7, NS2, NS3, NS4A, NS4B, NS5A, and NS5B), all of which are critical for viral replication. Key functions of these non-structural proteins include viral RNA capping, protease activity for polyprotein processing, helicase activity, and the formation of membrane-bound replication complexes [188].

HCV infection promotes HCC progression by increasing TGF-β signaling, which induces EMT [189,190,191,192]. HCV core protein increases TGF-β expression and shifts its role from a tumor suppressor to a tumorigenic factor, thereby driving metastasis [189] (Table 1).

HCV glycoproteins induce EMT in neoplastic hepatocytes by disrupting tight and adherens junctions and inducing Snail and Twist expression [193] (Table 1). HCV nonstructural protein NS5A activates Twist2 expression, leading to liver cell depolarization and EMT induction [194]. The HCV NS5A protein also activates Wnt/β-catenin signaling, which reduces E-cadherin expression and induces EMT in infected liver cells [195,196].

Reduction in E-cadherin expression and upregulation of multiple mesenchymal markers, including vimentin, N-cadherin, Stat3, Snail1, Slug, Twist1, and Zeb1, were detected in HCV-infected liver cancer tissues [197]. Of note, Xing and colleagues [198] reported that increased vimentin expression was also critical for cell-to-cell transmission of HCV. These results suggest that virus-induced EMT may also promote viral spread in HCC.

Infection of primary hepatocytes with HCV genotype 1a or 2a induced EMT by increasing vimentin, Snail, Slug, and Twist expression, and promoting the loss of E-cadherin expression [199]. Expression of Snail and Twist was also upregulated in chronically HCV-infected liver biopsy specimens, indicating ongoing EMT in virus-infected liver [199].

Expression of HCV core protein in normal hepatocytes and HCC tumor cells results in decreased miR-30c and miR-203a expression, leading to Snail1 and Snail2 upregulation and EMT induction [200]. HCV infection also results in increased miR-375 expression, promoting EMT and cell invasion [185,201,202]. Pradhan and colleagues [201] reported upregulation of exosome-associated miR-375 in HCV-infected patients with HCC and cirrhosis, as well as in HCV-infected Huh7.5 hepatoma cells. Depletion of miR-375 blocked HCV-induced cell migration and proliferation, suggesting a supportive role for miR-375 in HCV-induced EMT [201].

Upregulation of miR-21 was observed in cells from HCV-infected liver biopsies and sera from patients with chronic disease and HCC with poor prognosis [203,204,205]. Furthermore, miR-21 activates TGF-β signaling and upregulates SMAD7 [203]. Collectively, these results suggest that miR-21 might be involved in EMT induction in HCV-infected HCC cells.

### 3.5. Kaposi’s Sarcoma-Associated Herpesvirus

Kaposi’s sarcoma-associated herpesvirus (KSHV), also known as human herpesvirus 8 (HHV8), is an etiological agent of Kaposi sarcoma (KS) [206]. KS is an AIDS-defining tumor with uncontrolled growth of endothelial cells with abnormal angiogenesis [207]. KS may develop on the skin and/or oral/intestinal mucosa and can spread to lymph nodes and lungs [207].

KSHV-infected microvascular endothelial cells lose the capacity to express vascular endothelial cell markers (e.g., VE-cadherin, CD31, CD34, CD36, vascular endothelial growth factor receptor-3, and vascular endothelial growth factor C) and acquire expression of mesenchymal markers (fibroblast-specific protein-1, alpha 2 smooth muscle actin, type I/III collagen, vimentin, and N-cadherin), indicative of EndMT with invasive and migratory properties [208,209,210,211,212,213,214,215,216] (Table 1). KSHV-activated Notch-induced transcription factors Slug and Zeb1 were identified as critical for KSHV-induced EndMT [208,209]. The TGFβ signaling pathway is also required for EndMT induction [217]. However, KSHV does not activate the TGFβ signaling pathway in endothelial cells [208,209].

KSHV also infects epithelial cells and induces an EMT in MDA-MB-231T breast cancer cells, with increased cell migration, invasion, and survival driven by the viral latency-associated nuclear antigen (LANA) [218]. LANA induced EMT by upregulation of vimentin and transcription factor TCF8/ZEB1 and downregulation of tight junction protein ZO1 and adhesion protein E-cadherin. LANA co-localized with Zeb1, suggesting its direct interaction with a critical EMT contributor [218].

KSHV-induced EMT was detected in telomerase (hTERT)-immortalized normal oral gingival keratinocytes infected with latent KSHV [6]. KSHV-infected cells show downregulation of E-cadherin and upregulation of vimentin expression and higher migration and invasion compared to uninfected cells. These data suggest that KSHV-induced EMT in oral neoplastic cells may contribute to their invasiveness.

## 4. Non-Oncogenic Viruses

### 4.1. Human Cytomegalovirus

HCMV is an important human pathogen of the *Herpesviridae* family that infects salivary gland epithelial cells and subsequently spreads throughout the body, establishing latency in the myeloid progenitor cells [219,220,221]. HCMV is associated with opportunistic infection during HIV/acquired immunodeficiency syndrome (AIDS) [222]. It manifests as oral mucosal lesions, esophagitis, retinitis, pneumonia, hepatitis, gastrointestinal inflammation, or encephalopathy [223,224,225,226,227].

Accumulating evidence suggests that HCMV may promote neoplastic cell growth and invasiveness in various diseases, including colorectal cancer [13,14,15,16,228,229,230] and glioma [15,17,18,19,20,231,232,233,234,235,236].

HCMV induces EMT in glioma cells by activating the receptor-interacting protein kinase 2 and NF-κB signaling pathway, downregulating E-cadherin expression, and upregulating N-cadherin and vimentin expression [20,237] (Table 1). The HCMV transactivator proteins IE1 (p72) and IE2 (p86) are highly expressed in glioblastoma tumors and promote stemness in cancer cells that have undergone EMT via upregulation of CD133, nestin, and Sox2 [18]. HCMV IE proteins are frequently co-expressed with the stem cell markers CD133 and nestin [18,238].

HCMV infection is associated with EMT and enhanced invasiveness of colorectal cancer cells with stem cell phenotypes [13,14]. HCMV infection of neoplastic epithelial cells may be critical for the TGF-β-dependent induction of cancer-associated EMT and the suppression of anticancer immune responses [36,239,240].

HCMV infection has also been associated with bile duct disorders, including sclerosing cholangitis, biliary atresia, and post-transplant biliary complications [241]. Ye and colleagues [241] showed that HCMV infection induced EMT via TGF-β activation in a cholangiocyte-like cell organoid model.

Results of transcriptomic analysis of mammary epithelial cells infected with the clinical oncogenic HCMV strain (MW980585) revealed upregulation of genes involved in cell survival, proliferation, and EMT, suggesting that HCMV infection may promote cancer cell migration and invasion via EMT-associated mechanisms [242].

### 4.2. Human Immunodeficiency Virus

HIV-1 is a non-oncogenic retrovirus that infects CD4^+^ T lymphocytes, monocytes/macrophages, and dendritic cells. HIV-1 is the etiologic agent in AIDS, a disorder characterized by progressive immune dysfunction and the development of opportunistic infections, cancer, and other disorders. HIV-susceptible CD4^+^ T lymphocytes, monocytes/macrophages, and dendritic cells are localized in all organs, including genital, intestinal, and oropharyngeal mucosal epithelium [243,244,245,246,247,248,249,250,251].

In vitro experiments have shown that prolonged interaction of cell-free HIV-1 virions, as well as viral envelope and transactivator proteins gp120 and tat, respectively, results in EMT of tonsil, cervical, and foreskin epithelial cells [8] (Table 1). Interaction of cell-free virions as well as gp120 and tat proteins with epithelial cells substantially reduced E-cadherin expression and activated vimentin and N-cadherin expression. HIV gp120- and tat-induced EMT was mediated by SMAD2 phosphorylation and activation of transcription factors Slug, Snail, Twist1 and Zeb1 [8].

Activation of TGF-β and MAPK signaling by gp120, tat, and cell-free HIV virions revealed the critical roles of these signaling pathways in EMT induction. Gp120- and Tat-induced EMT cells were highly migratory, which is one of the main features of mesenchymal cells. Inhibitors of TGF-β1 and MAPK signaling inhibited HIV-mediated induction of EMT, indicating that inactivation of these signaling pathways induces MET, restoring normalization of epithelial phenotype [8].

Because HIV is not an oncogenic virus, HIV-induced EMT in normal epithelial cells does not lead to a neoplastic process; rather, this may lead to depolarization of the epithelium, disruption of cell junctions, and reduction in barrier function [7,8,251,252,253,254,255].

HIV gp120 and tat also induce EMT in neoplastic oral, genital, and lung cells [7,10,256]. HIV tat-induced EMT and cell migration and metastasis in non-small cell lung cancer were reduced in response to the loss of function of tat-interacting protein 30 (TIP30), which is a tumor suppressor protein and specific cofactor that enhances HIV-1 Tat-activated transcription [257,258]. In normal cells, TIP30 is highly expressed; its expression is reduced or lost in neoplastic diseases, leading to the suggestion that TIP30 may serve as a prognostic marker for tumor metastasis [258,259,260]. HIV Tat promotes the nuclear translocation of Snail, leading to EMT induction. The interaction of tat with TIP30 blocked TIP30 binding to importin-β [256], which is a nuclear transport receptor and required for the nuclear translocation of Snail [261].

**Table 1:** Interactions of human pathogenic viruses with cellular proteins that regulate the EMT program.

Virus	Cellular Proteins and Signaling Pathways	Potential Mechanisms of EMT Induction	References
Human papillomavirus (HPV)	EGFR, MAPK, PI3K/Akt, FGF-2/4, Jagged1, FGFR2b and FGFR2c	High-risk HPV oncoproteins E6, E7, and E5 activate TGF-β and MAPK signaling and upregulate Twist, Zeb, and Slug transcription factors, thereby promoting EMT.	[99,102,103,104,105,106,107,108]
		HPV-16 and HPV-18 infection in cervical cancer cells induces EMT by upregulating the expression of the calcium signaling mediator transient receptor potential canonical-3 (TRPC3), which activates MAPK.	[110]
		The high-risk HPV E6 protein activates JNK and EGFR signaling and induces EMT by upregulating Slug and vimentin.	[109]
		High-risk HPV-16 E5 and E6/E7 proteins activate MAPK and PI3K/Akt signaling and upregulate Zeb, Snail, and Twist transcription factors, thereby inducing EMT.	[111,112,113,117,118,121]
		High-risk HPV E6/E7 upregulate MMP-2, MMP-9, and MT1-MMP, which cleave E-cadherin and digest the extracellular matrix, thereby promoting EMT.	[128,129,130,132,133,134]
		HPV-16 E5 downregulates the epithelial cell-expressed FGRF2b receptor and upregulates the mesenchymal cell-expressed FGFR2c receptor, thereby promoting EMT.	[142]
		HPV E6/E7 upregulate both PD-L1 and promote EMT to facilitate immune evasion and cancer cell invasion.	[121,126,127]
Epstein–Barr virus (EBV)	TGF-β, NF-ĸB, ERK-MAPK, PI3K/Akt, Snail, Twist, MMP-9, Wnt/β-catenin, miR-200	EBV LMP1 activates signaling pathways, including TGF-β, NF-κB, ERK-MAPK, and PI3K/Akt, leading to induction of EMT.	[147,148,149]
		EBV LMP1 activates upregulation of Snail and Twist and induces MMP-9 expression to generate EMT.	[150,151,152,153]
		EBV LMP2A induces EMT by activating PI3K/Akt signaling.	[155]
		Co-expression of both EBNA1 and EBNA3C in cancer cells can induce EMT by upregulating Slug, Snail, Zeb1, and vimentin	[158]
		EBV EBNA1 promotes the EMT program by activating TGF-β signaling and inhibiting miR-200.	[165]
		EBV infection-induced downregulation of miRNA 200 leads to activation of Wnt/β-catenin signaling, thereby promoting EMT.	[159,160,161,164]
		EBV-associated downregulation of miR-200a and miR-200b in gastric carcinoma cells induces EMT by activation of Wnt/β-catenin and c-Jun/Slug signaling, upregulation of ZEB1/ZEB2.	[159,160,161,162,163,164]
		EBV EBNA1 induces EMT, promoting metastasis and invasion of NPC cells by inhibiting miR200 and activating TGF-β signaling, upregulation of Zeb1 and Zeb2.	[152,157]
		EBV LMP1 simultaneously increases PD-L1 expression and promotes EMT in neoplastic cells.	[165]
Hepatitis B virus (HBV)	PI3K/Akt/GSK3β, TGF-β, JNK, c-Src, Snail, E12/E47 transcription repressor, miR-520c-3p, miR-148a, miR-122, miR-15a, Smad7	HBV HBx protein promotes EMT by activating PI3K/Akt, JNK, and c-Src signaling pathways that downregulate E-cadherin and elevate vimentin expression.	[172,173,174]
		The HBV HBx protein inhibits E-cadherin expression and induces EMT by increasing the expression of transcriptional repressors E12/E47 and Snail.	[175]
		HBV HBx activates the PI3K/Akt/GSK3β signaling and c-Src kinase to activate vimentin expression and induce EMT.	[172,173,174]
		HBx and HBc proteins induce EMT by activation of JNK pathway.	[176]
		HBV infection induces EMT by upregulating oncogenic miR-520c-3p and downregulating tumor-suppressive miRNAs, including miR-148a and miR-122.	[178,179,180]
		HBV infection suppresses miR-15a, leading to upregulation of Smad7 and induction of EMT.	[182,183]
Hepatitis C virus (HCV)	TGF-β, Snail, Twist 2, Wnt/β-catenin, miR-30c, miR-203a, miR-375	HCV Core proteins increases TGF-β expression in neoplastic liver cells, thereby leading to EMT.	[189]
		HCV glycoproteins induce EMT by disrupting tight and adherens junctions and activating the transcription factors Snail and Twist.	[193]
		HCV nonstructural protein NS5A activates TWIST2 expression, leading to depolarization of liver cells and induction of EMT.	[194]
		The HCV NS5A protein activates Wnt/β-catenin signaling, inhibits E-cadherin expression, and induces EMT in virus-infected liver cells.	[195,196]
		HCV infection of hepatocytes induced EMT by increasing vimentin, Snail, Slug, and Twist expression, and promoting the loss of E-cadherin expression.	[199]
		Expression of HCV Core protein inhibits miR-30c and miR-203a expression, leading to Snail upregulation and induction of EMT.	[200]
		HCV infection increases miR-375 expression, thereby promoting EMT and cell invasion.	[185,201,202]
		HCV infection upregulates miR21, which activates TGF-β signaling and upregulates SMAD7 leading to EMT induction.	[203,204,205].
Kaposi’s sarcoma-associated herpes virus (KSHV)	VE-cadherin, E-cadherin, ZO-1, CD31, CD34, CD36, vascular endothelial growth factor receptor-3, and vascular endothelial growth factor C, Notch and TGF-β signaling, Slug, Zeb1, TCF8/ZEB1	KSHV infection induces EndMT in microvascular endothelial cells by inhibiting expression of VE-cadherin, CD31, CD34, CD36, vascular endothelial growth factor receptor-3, and vascular endothelial growth factor C.	[208,209,210,211,212,213,214,215,216]
		KSHV induces EndMT in endothelial cells by activation of Notch signaling and Slug and Zeb1 expression.	[208,209]
		KSHV protein LANA induces EMT in epithelial cells by upregulation of vimentin and transcription factor TCF8/ZEB1 and downregulation of tight junction protein ZO1 and adhesion protein E-cadherin.	[218]
		KSHV induces EMT in epithelial cells by upregulating vimentin and downregulating E-cadherin expression.	[6]
Human cytomegalovirus (HCMV)	Receptor-interacting protein kinase 2, NF-κB, TGF-β, Wnt/β-catenin	HCMV induces EMT in glioma cells by activating receptor-interacting protein kinase 2 and NF-κB signaling pathway, downregulating E-cadherin expression, and upregulating N-cadherin and vimentin.	[20,237]
		The HCMV transactivator proteins IE1 (p72) and IE2 (p86) promote stemness in glioblastoma cancer cells that have undergone EMT.	[18]
		In colorectal cancer cells, HCMV infection and the presence of IE proteins upregulate the Wnt/β-catenin signaling pathway, thereby facilitating cell migration and metastasis.	[13,14]
		HCMV infection induced EMT in a cholangiocyte-like cell organoid mode by TGF-β activation.	[241]
Human Immunodeficiency Virus (HIV)	TGF-β, MAPK, Snail, Twist1, Zeb1, Wnt/β-catenin, CD44, CD133	HIV gp120- and tat-induced EMT in normal tonsil, cervical and foreskin epithelial cells is mediated by SMAD2 phosphorylation and activation of TGF-b, MAPK, and transcription factors Slug, Snail, Twist1, and Zeb1.	[8]
		HIV-1 tat and gp120 activate TGF-β and MAPK signaling and promote EMT in HPV-16 infected neoplastic cervical CaSki and anal AKC-2 epithelial cells.	[7,10,256]
		HIV-promoted EMT in CaSki and AKC-2 cells that express cancer stem cell markers CD44 and CD133.	[7]
		Treatment of HPV-16-infected cervical cancer SiHa cells with HIV-1 Tat and gp120 induced EMT and cell migration and invasion by activating the Wnt/β-catenin pathway.	[7,10,256]
		More EMT cells were detected in cervical cancer cells that were coinfected with HIV and HPV.	[10]
		HIV-1 tat induces EMT in lung cancer cells.	[256]

## 5. Viral Coinfection and Its Role in EMT Induction

Viral coinfections, including oncogenic/oncogenic and oncogenic/non-oncogenic viruses, can significantly accelerate cancer progression by inducing the EMT, leading neoplastic cells to become more invasive and metastatic. Combined viral infections may activate and amplify TGF-β, MAPK, and other EMT-relevant signaling pathways, and upregulate Snail, ZEB, and Twist family transcription factor complexes, showing synergistic and/or additive effects on the EMT program [22,262,263].

### 5.1. The Role of HIV and HPV Coinfection in EMT Induction

People living with HIV (PLWH) are at higher risk for developing HPV-associated anogenital and/or oropharyngeal carcinomas [264,265,266,267,268]. Despite the availability of a U.S. Food and Drug Administration-approved anti-HPV vaccine, the incidence of HIV- and HPV-associated anogenital and oropharyngeal carcinomas is expected to continue to rise over the coming decades because this strategy is not highly effective at preventing disease in HIV-infected men and women with multiple previous exposures [269].

The incidence of HPV-associated oropharyngeal, anal, and cervical cancer is 6-, 80-, and 22-times higher, respectively, in HIV-infected individuals than in uninfected men and women [270,271,272,273,274]. This suggests that HIV and HPV coinfection may play a critical role in the progression of HPV malignancy via their capacity to induce EMT. HIV-induced EMT has been demonstrated in HPV-16-positive neoplastic cells [7,9]. HPV-16-positive anal AKC-2 and cervical CaSki epithelial cells that undergo prolonged exposure to cell-free HIV-1 virions or to HIV-1 viral proteins gp120 and tat exhibit an EMT phenotype and increased invasiveness [7]. EMT induced by gp120 and Tat led to the detachment of poorly adherent cells from the culture substratum; these cells remained capable of reattaching and, upon reattachment, co-expressed both E-cadherin and vimentin, indicative of an intermediate stage of EMT. The reattached cells also expressed stem cell markers CD133 and CD44, which may play a critical role in cancer cell invasion and metastasis. Inhibition of TGF-β1 and MAPK signaling and vimentin expression, and restoration of E-cadherin expression, reduced HIV-induced EMT and the invasive activity of HPV-16-positive anal and cervical epithelial cells [7,9].

Treatment of HPV-16-infected cervical cancer SiHa cells with HIV-1 Tat and gp120 induced EMT and cell migration and invasion by activating the Wnt/β-catenin pathway [10]. Transcriptome analysis revealed that Tat/gp120-treated cells responded by upregulating keratin 17 and downregulating GSK3b, thereby promoting increases in vimentin and decreases in E-cadherin expression (i.e., EMT induction) [10]. Cervical cancer cells from HIV-positive patients had lower E-cadherin and cytokeratin, and higher N-cadherin and vimentin levels than cells from HIV-negative patients [10]. HIV tat-induced EMT was also detected in HPV-negative oral and lung cancer cells, suggesting that HIV-associated EMT may occur in other neoplastic diseases that are not initiated by oncogenic viruses [7,256].

There has been no apparent decline in the incidence of HPV-associated cancers in the HIV-infected population despite the widespread use of combination anti-retroviral therapy (cART). If HIV infection is a biologically relevant contributor to EMT in the HPV-infected neoplasia, then HIV proteins should be present in the mucosal environment of cART-treated HIV/HPV coinfected individuals. Actively replicating HIV virions can be detected in multiple organs and tissues of individuals receiving cART, including the oral, gut, anal, and cervix [243,244,245,246,247,251]. Resident memory CD4^+^ T lymphocytes in various tissues, including the lower genital tract, serve as a persistent HIV reservoir under cART [248,249,250]. HIV-infected tissue macrophages also play critical roles in maintaining viral latency and generating persistent reservoirs in patients with AIDS receiving maintenance cART [251,275,276,277,278,279,280,281,282].

Drugs included in cART have only a limited ability to penetrate solid tissues, including lymph nodes and epithelial tissues [283,284,285]. Therefore, HIV-1 replication may persist in these drug-inaccessible environments and lead to the expression, release, and spread of tat and gp120 within surrounding tissues and organs. Evaluation of cervicovaginal secretions of HIV-1 in ART-treated women with low to undetectable plasma viral loads suggested that there may be a cohort of local intramucosal HIV-1-infected cells that remain capable of viral production because of limited drug accessibility [286,287,288,289,290]. This hypothesis might also explain the persistent detection of HIV-1 in the semen of ART-treated infected men with undetectable viral loads in circulation [291]. These findings suggest that ART-inaccessible tissues, including oral and genital mucosal epithelium, might serve as a reservoir for HIV-1 [285], and that these cells might continuously release Tat and gp120 into the surrounding environment. Since HPV infection and carcinogenesis occur in the oral and genital mucosal epithelium, and HIV replication and EMT induction in these HPV-infected neoplastic epithelium may accelerate HPV malignancy.

Accumulating evidence indicates that HIV Tat expression and secretion occur regardless of cART treatment, showing the role of Tat in EMT induction in HPV-infected individuals. In the pre-cART era, serum Tat concentrations in PLWH typically ranged from 0.1 to 40 ng/mL, and sometimes reached 250–550 ng/mL [292,293,294,295,296]. HIV-1 Tat was also detected at concentrations of 0–14 ng/mL in the serum in PLWH who achieved viral suppression with cART [297]. Tat was reported in 25% of cART-treated individuals [297]. Serum concentrations of tat during early cART and late cART of 2–40 ng/mL and 0.2–9 ng/mL, respectively, were reported [292,294]. HIV-1 Tat was also detected in the blood–cerebrospinal fluid in 37% of PLWH maintained on a long cART regimen [296,298,299], at concentrations of 0.2–6.5 ng/mL, and functional Tat was detected in exosomes from 34.4% of these individuals [298]. Moreover, Tat was detected in the saliva in both cART-treated and untreated individuals [251].

Results from experiments performed in vitro revealed that Tat is expressed and secreted by HIV-infected cells treated with protease inhibitors that block viral replication [296,298,300]. Tat/TAR-containing exosomes can be endocytosed by HIV-1-infected cells that contain an inactive (latent) provirus [298]. Internalized Tat/TAR may then reactivate the latent provirus via the induction of host signaling pathways such as those involving NF-κB, as well as transactivation of the HIV-1 long terminal repeat, thus potentially explaining the Tat expression detected in PLWH maintained on cART [298]. Tat expression in PLWH treated with cART might also result from ongoing abortive viral transcription that promotes expression of viral early genes, including Tat [301]. For example, Tat expression has been detected in non-productive latent HIV-1-infected astrocytes [302,303]; therefore, Tat might be expressed in cells infected with latent viruses in general [304]. Tat might also be expressed in cells that support spontaneous transcription and translation of HIV-1 that emerges despite cART [305].

Taken together, current evidence suggests that HIV-infected memory CD4^+^ T lymphocytes, dendritic cells, and macrophages in PLWH treated with cART express and secrete Tat and gp120, both of which might interact with HPV-infected neoplastic epithelial cells and subsequently induce EMT, thereby accelerating their invasion (Figure 4). Thus, although HIV-1 is not an oncogenic virus, it plays an oncomodulatory role by accelerating HPV-associated malignancy by promoting EMT [7,10].

### 5.2. HIV-Induced Endothelial–Mesenchymal Transition in KSHV-Infected Endothelial Cells Promotes Invasion of Kaposi Sarcoma

Kaposi Sarcoma is the most common cancer in untreated PLWH. While KSHV initiates the KS neoplastic process, HIV acts as a cofactor that promotes its progression through immunosuppression and direct interaction with tumor cells. The extracellular HIV protein Tat plays a critical role in KS pathogenesis by synergizing with KSHV lytic proteins and inducing EndMT, promoting cell proliferation, angiogenesis, and migration [293,306,307,308,309,310,311,312,313,314,315,316,317]. HIV Tat-mediated interactions with endothelial cells lead to increased expression and signaling of the KSHV G protein-coupled receptor (vGPCR), promoting viral infection and tumorigenicity [318,319,320,321]. HIV Tat also promotes KSHV IL-6 (vIL-6)- and K1-induced angiogenesis [317,322]. The HIV Tat-mediated increased oncogenic activity of KSHV proteins occurs primarily in response to HIV Tat-associated regulation/modulation of the PI3K/Akt signaling pathway [317,320,323]. Tat may also be involved in the expression of miRNAs, which increase angiogenesis and thus accelerate KSHV-associated tumorigenesis [322].

In the pre-cART era, KS was approximately 20–50-fold more common in persons with HIV/AIDS than in the general population [324,325]. In PLWH, cART treatment markedly reduced the incidence of KS [326,327]. However, recent findings suggest that the risk of KS is increasing in PLWH despite normalization of the CD4^+^ count and lack of HIV viremia [328,329,330,331]. Secretion of Tat in PLWH undergoing cART may contribute to the development of KS via Tat-induced EndMT and migration of KSHV-infected endothelial cells. Moreover, HIV-1 Tat-induced activation of the TGF-β signaling pathway might synergize with KSHV-induced Notch signaling, thereby promoting EndMT and accelerating the migration and invasion of neoplastic endothelial cells. Increasing KS in PLWH with cART suggests the need for additional research on the pathogenesis of HHV-8 [332].

HIV and KSHV interact with one another to induce EndMT, thereby contributing to the extreme aggressiveness of HIV/AIDS-associated Kaposi’s sarcoma. While KSHV is the direct etiological agent of KS, HIV Tat works indirectly by amplifying TGF-β, MAPK, and NF-κB signaling to promote neoplastic endothelial cell proliferation, migration, and invasion. The inflammatory and viral milieu of HIV/AIDS promotes neoplastic cellular plasticity, facilitating transitions between endothelial and mesenchymal phenotypes, and creating the characteristic heterogeneous cancer spindle cell masses of HIV/AIDS-associated KS.

### 5.3. HIV, HCMV, and HPV Coinfection Promote HPV-Associated Malignancy

HIV, HCMV, and HPV coinfection is common in PLWH [333]. Collectively, these viruses may activate TGF-β, MAPK, and PI3K/Akt signaling pathways and upregulate EMT-related transcription factors. This may lead to additive and/or synergistic amplification of EMT in HPV-infected oral and genital epithelial cells and accelerate the progression of HPV-associated neoplastic disease.

Although the majority of the human population is infected with HCMV (40–95%), the virus is typically latent under normal immune surveillance in healthy individuals [334]. However, in PLWH with HIV/AIDS-associated immune dysfunction, HCMV can be reactivated and consequently infect epithelial and other cells [335,336,337,338,339,340,341,342,343,344,345,346,347,348] and induce TGF-β expression and secretion [349,350,351,352,353,354,355]. The results of in vitro studies revealed that coinfection of tonsil cells with HCMV and HIV-1 synergistically promotes the infection and spread of both viruses [255].

HCMV infection may occur in cervical tissues that have already been infected with high-risk human papillomavirus (HPV) [333,356,357,358,359,360]. HCMV co-infection with HPV16 or HPV18 is observed more frequently in high-grade cervical lesions and invasive carcinoma than in normal cervical tissues [333,361,362,363]. Paradowska and colleagues [364] reported detection of HCMV and HPV DNA in 70% and 74% of ovarian epithelial cancer tissues, respectively, and coinfection with both viruses in almost two-thirds of the pathological tissues analyzed. HCMV has also been detected in colorectal cancer tissues from both HIV-positive and HIV-negative patients [365,366,367,368].

HCMV, HIV, and HPV infections upregulate MMPs, which are critical for activating the TGF-signaling pathway. Latent TGF-β is tethered to the extracellular matrix by the Latent TGF-β Binding Protein (LTBP). MMPs digest the surrounding matrix proteins, including collagen and fibronectin, thereby releasing the entire latent complex, rendering it accessible to other local activation mechanisms [369,370,371]. Furthermore, MMPs activate TGF-b by cleaving the latency-associated peptide (LAP) portion of the large latent complex and releasing the active cytokine so it can bind to its cell-surface receptors [371,372]. Numerous proteases, including the MMP2 and MMP9, play critical roles in the activation of LLC of TGF-β [349,371,373,374,375,376,377]. HCMV IE1/2 proteins have been found to increase MMP2 expression in kidney epithelial cells [349,378]. HIV envelope protein gp120 also induces MMP9 expression [254] and thus contributes to the activation of TGF-β signaling and the induction of EMT [7,8]. HPV-16 E6 and E7 oncoproteins also promote increased expression of MMP2 and MMP9 [131,132,133,134]. Thus, HIV, HCMV, and HIV can contribute to the activation of TGF-β signaling, promoting EMT induction.

HIV-1-induced EMT may also promote epithelial neoplasia associated with other oncogenic viruses. For example, HIV-HCV co-infection is detected quite frequently; ~30% of HIV-infected individuals may be coinfected with HCV [379,380,381,382]. Primary liver cancer is more aggressive in patients with HIV-HCV coinfection than in those with HCV infection alone [383]. HIV-1 Tat-induced EMT may develop in liver epithelial cells, thus accelerating HCV malignancy in coinfected individuals.

HIV and HBV coinfection occurs in ~5–20% of PLWH worldwide [384]. Since both viruses can induce EMT, coinfection may accelerate EMT induction in HBV-infected neoplastic cells, leading to the progression of cirrhosis and liver cancer.

Worldwide prevalence of HBV and HCV coinfection has been estimated as high as 15% [385,386]. Coinfection may additively and/or synergistically induce EMT, accelerating liver fibrosis, cirrhosis, and the progression of HCC. During HBV and HCV co-infection, HBx and HCV core proteins may induce EMT by downregulation of E-cadherin and upregulation of N-cadherin, Snail, Twist, and vimentin expression via DNA methylation [387].

## 6. Conclusions and Future Prospects

Accumulating evidence suggests that several oncogenic and non-oncogenic viruses induce EMT, a state in which neoplastic epithelial cells lose polarity and cell-to-cell adhesion, thereby acquiring migratory and invasive properties. This cellular reprogramming promotes cancer progression and tissue fibrosis. Oncogenic HPV, EBV, HBV, HCV, and KSHV, and non-oncogenic HCMV and HIV, induce EMT in neoplastic cells by triggering a variety of mechanisms. Activation of the TGF-β, MAPK, and Wnt/β-catenin signaling pathways is a primary trigger for EMT used by many of these viruses, leading to downregulation of the epithelial marker E-cadherin and upregulation of mesenchymal markers vimentin and N-cadherin. These viruses also upregulate master EMT transcription factors such as Snail, Slug, Zeb, and Twist, which suppress epithelial genes while promoting the expression of mesenchymal markers.

Coinfection of neoplastic cells with two or more oncogenic viruses or oncogenic with non-oncogenic viruses may synergistically and/or additively amplify EMT signals and transcription factor expression, thereby accelerating invasion and metastasis of neoplastic cells. Understanding the molecular mechanisms underlying the synergistic/additive effects of viruses will open new avenues for interfering with these interactions and thus preventing EMT.

Terminating EMT and inducing MET are major therapeutic goals for preventing cancer metastasis and organ fibrosis. Agents that induce MET by suppressing vimentin expression and restoring E-cadherin expression in targeted neoplastic cells may lead to new approaches for treating invasive cancers. Ongoing studies highlight the feasibility of terminating EMT via MET induction using specific compounds that inhibit vimentin and restore E-cadherin expression. For example, the flavonolignan silibinin, an organosulfur compound of milk thistle, can be used to inhibit vimentin expression [388,389]. Similarly, sulforaphane, a compound that inhibits Zeb-1, a repressor of E-cadherin expression, might be introduced to restore E-cadherin expression [390,391]. The plant protein α-solanine might also be developed as a pharmaceutical to inhibit vimentin expression and upregulate E-cadherin [392]. The histone D-acetylase inhibitor vorinostat might also be explored as a means to terminate EMT in cancer cells by increasing E-cadherin expression [393].

Further studies are needed to rapidly identify and investigate EMT status in neoplastic tissue environments in the clinical setting. High-tech approaches, including single-cell RNA sequencing of neoplastic lesions, may identify EMT in the tumor microenvironment by measuring cell-specific gene expression changes. This may uncover transitional or hybrid states of EMT in which cells simultaneously express both epithelial and mesenchymal programs. It is highly important to develop targeted drugs that inhibit the actions of the master regulatory transcription factors leading to EMT in neoplastic cells, including Snail, Slug, Zeb, and Twist, thereby removing the genetic blocks on epithelial genes (such as E-cadherin). Using a combination of specific inhibitors of EMT signaling pathways, including TGF-β, MAPK, and Wnt/β-catenin in neoplastic cells, will block signals that promote the mesenchymal state.

Nanoparticles are a highly promising strategy for delivering EMT inhibitors directly into neoplastic lesions [394,395]. Critical approaches currently under consideration include using lipid, polymeric, or inorganic nanocarriers to deliver specific therapeutics designed to block EMT signaling pathways and induce MET, thereby preventing tumor metastasis [101,395]. Targeted delivery of inhibitors of vimentin, EMT transcription factors, and signaling pathways into neoplastic lesions with nanocarriers may also increase their efficacy. The application of highly efficient EMT nanotechnology approaches, in combination with chemotherapy and/or immunotherapy, may inhibit cancer cell invasion and promote the regression of neoplastic processes.

## Figures and Tables

**Figure 1:**
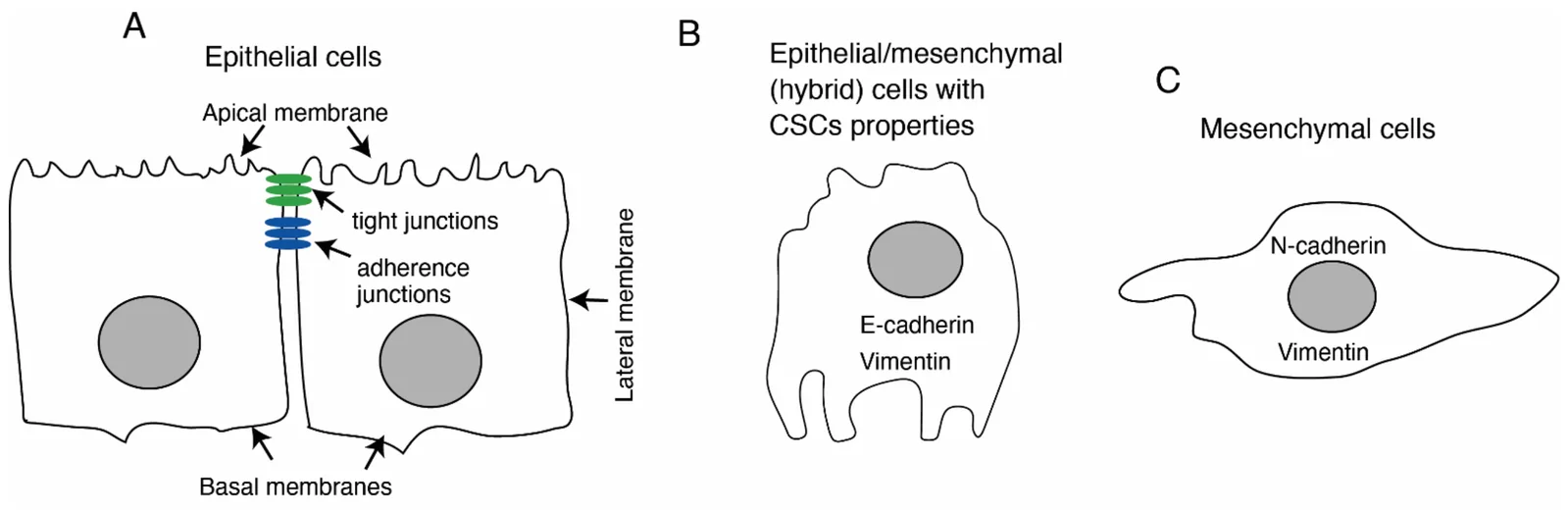
Model of EMT. (**A**) Epithelial cells have an apicobasal polarized organization with well-developed tight and adherens junctions and distinct apical and basolateral membranes. While undergoing EMT, epithelial cells lose their apicobasal polarity and intercellular junctions. This loss of spatial organization and adhesion allows them to reorganize their cytoskeleton, gain migratory capabilities, and transform into mobile, invasive mesenchymal cells. (**B**) In the intermediate stage of EMT, epithelial cells still express both epithelial (E-cadherin) and mesenchymal (vimentin) markers, exhibiting a hybrid EMT phenotype that may harbor cancer stem cell features. (**C**) In the terminal stage of EMT, cells lose epithelial markers and express only mesenchymal markers, which allows them to aggressively migrate within the tumor microenvironment and promote tumor invasion and metastasis.

**Figure 2:**
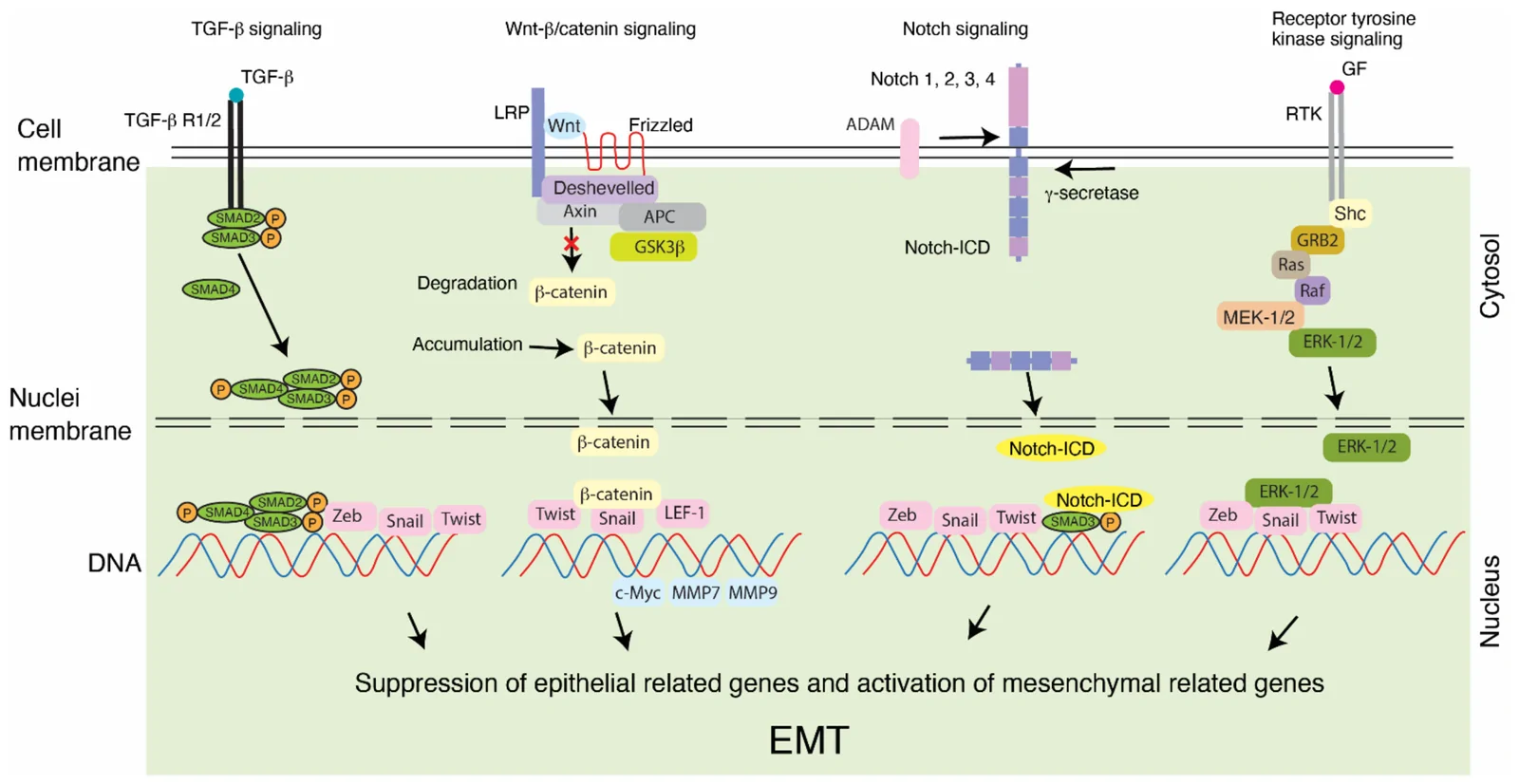
Major signaling pathways involved in the regulation of EMT. TGF-β signaling: The interaction of TGF-β with its receptors (TGF-β R1/2) induces phosphorylation of SMAD1, SMAD2, and SMAD3, leading to complex formation. The complex is translocated into the nucleus, where it activates transcription factors Snail, Twist, and Zeb. This leads to the repression of epithelial marker expression (e.g., E-cadherin) and the activation of mesenchymal marker expression (e.g., vimentin), thereby inducing EMT. Cells that have undergone EMT lose junctions and polarity and can migrate. WNT/β-catenin signaling: Wnt ligand binding to its receptor (Fizzled) activates WNT/β-catenin signaling, leading to the recruitment of coreceptor LRP5/6, and inhibition of disheveled-dependent GSK-3β kinase, which facilitates the accumulation of β-catenin in the cytoplasm and its nuclear translocation. In the nucleus, β-catenin binds to the transcription factors Snail, Twist, and Zeb, thereby inducing EMT. Notch signaling: The binding of Notch ligands to the Notch receptor leads to receptor activation and cleavage by a disintegrin and metalloproteinase (ADAM) and γ-secretase. The released Notch intracellular domain will translocate into the nucleus and bind to phosphorylated SMAD3, which activates additional transcription factors and induces EMT. Receptor tyrosine kinase signaling: Growth factor (FGF, PDGF, EGF, or IGF) binding to receptor tyrosine kinases activates the MAPK signaling pathway, leading to the translocation of ERK-1/2 into the nucleus, followed by increased expression of transcription factors Snail, Twist, and Zeb, and induction of EMT. Abbreviations: APC, adenomatous polyposis coli; GSK3*β*, glycogen synthase kinase3-*β*; MMP7, metalloproteinase 7; MMP9, metalloproteinase 9; LEF, lymphoid enhancer-binding factor; Notch ICD, notch intracellular domain; Shc, Src homology 2 domain-containing; GRB2, growth factor receptor-bound protein 2; MEK-1/2, mitogen-activated protein kinase 1 and 2; ERK 1/2, extracellular signal-regulated kinases 1 and 2.

**Figure 3:**
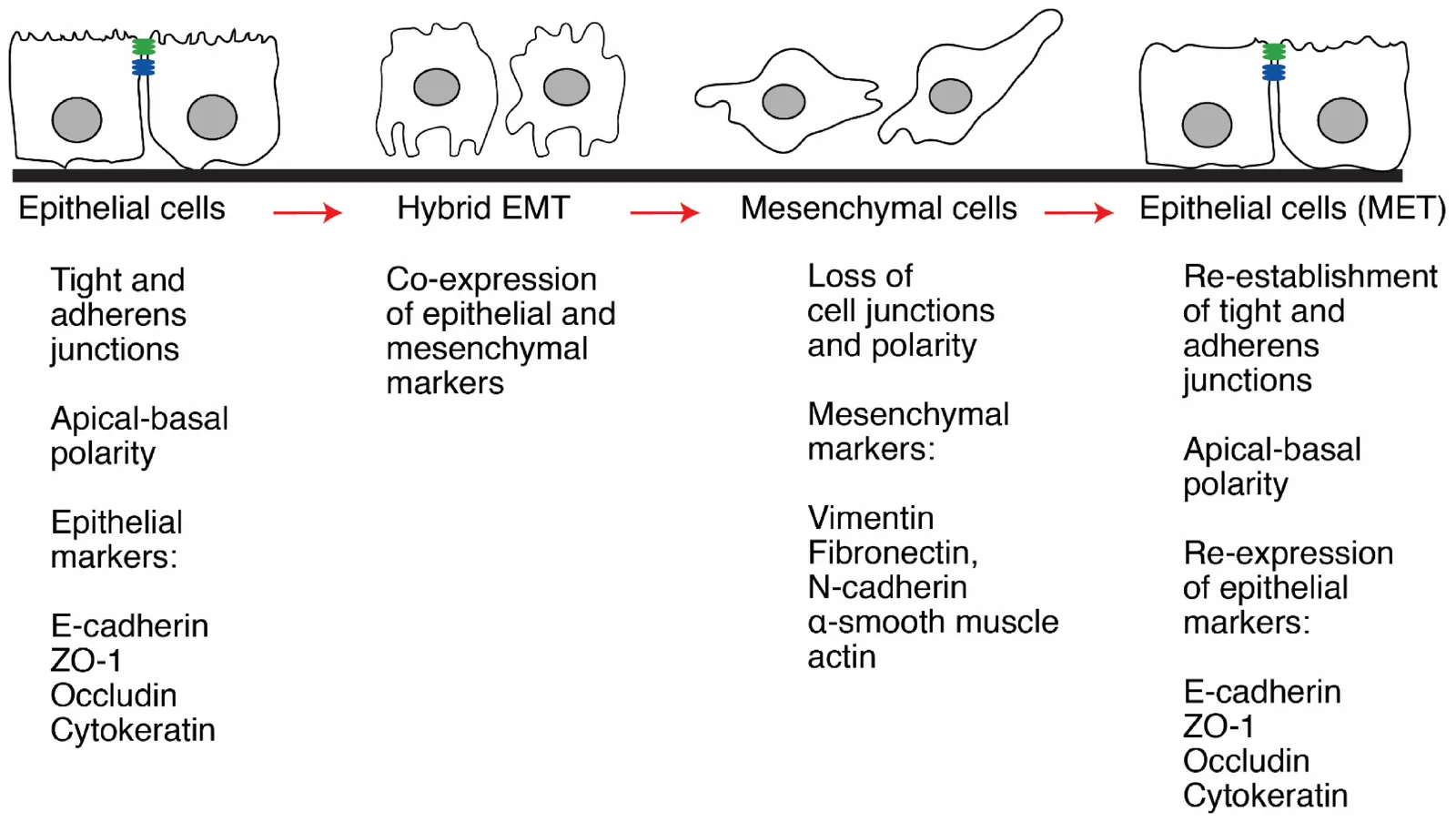
Model of EMT and MET cycling. In cancer, EMT and mesenchymal–epithelial transition (MET) form a dynamic, reversible cycle that drives tumor spread and metastasis. This cellular plasticity permits solid tumors to enter the blood circulation, invade surrounding tissues, and colonize distant organs.

**Figure 4:**
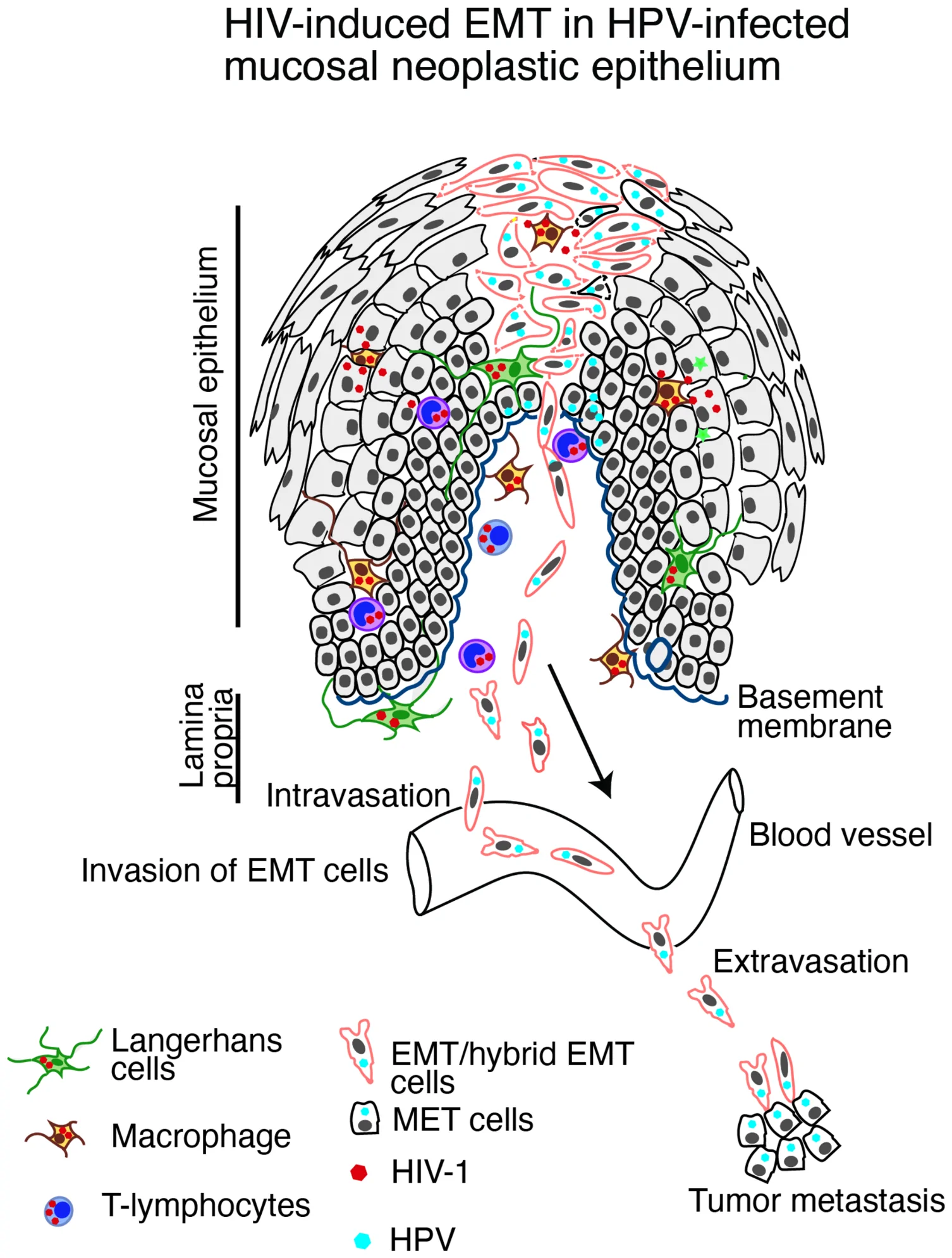
Model of HIV-associated EMT induction in the genital epithelium and its role in accelerating invasion of HPV-infected malignant cells. HIV-infected CD4^+^ T lymphocytes, dendritic/Langerhans cells, and macrophages migrate into high-risk HPV-infected neoplastic epithelial cells and secrete HIV tat and/or gp120. The HIV proteins induce EMT/hybrid EMT within the oral and anogenital epithelia. HPV proteins E6/E7 and E5 also induce EMT. Cells that have undergone EMT lose tight and adherens junctions and express N-cadherin, vimentin, and stem cell markers. HPV-infected neoplastic cells undergoing hybrid EMT can migrate through the basement membrane and subsequently enter blood vessels, disseminating throughout the body. Release of EMT/hybrid EMT cells from blood vessels into surrounding tissues/organs, and their reversion to a MET phenotype, leads to tumor metastasis and the formation of new cancer lesions.

## Data Availability

No new data were created or analyzed in this study. Data sharing is not applicable to this article.
